# Evaluation of Wisconsin and CaPTHUS Indices Usefulness for Predicting Monoglandular and Multiglandular Disease in Patients with Primary Hyperparathyroidism through the Analysis of a Single-Center Experience

**DOI:** 10.1155/2021/2040284

**Published:** 2021-10-11

**Authors:** Loredana De Pasquale, Eleonora Lori, Antonio Mario Bulfamante, Giovanni Felisati, Luca Castellani, Alberto Maria Saibene

**Affiliations:** ^1^Thyroid and Parathyroid Surgery Service (Head Dott. Loredana De Pasquale)-Otolaryngology Unit (Head: Professor Giovanni Felisati), ASST Santi Paolo e Carlo, Department of Health Sciences, Università Degli Studi di Milano, Via A. di Rudinì, 8, Milan 20142, Italy; ^2^Department of Surgical Science, “Sapienza” University of Rome, Rome 00161, Italy; ^3^Otolaryngology Unit (Head: Professor Giovanni Felisati), ASST Santi Paolo e Carlo, Department of Health Sciences, Università Degli Studi di Milano, Via A. di Rudinì, 8, Milan 20142, Italy

## Abstract

**Background:**

The main challenge for treating primary hyperparathyroidism (PHPT) is to understand if it is caused by a single adenoma (80–85% of the cases) or by a multiglandular disease (15–20%), both preoperatively and intraoperatively. For this reason, some preoperative scores were proposed in the literature, to perform focused parathyroidectomy, avoiding intraoperative parathormone assay (ioPTH). The most known are the CaPTHUS test and the Wisconsin index. We applied them to our experience.

**Methods:**

A retrospective cohort study on 462 patients referred for parathyroidectomy to Thyroid and Parathyroid Unit at Santi Paolo e Carlo Hospital, Milan, Italy, from 2011 to 2021. Only patients affected with benign PHPT and neck ultrasound performed at our institution were included. Both patients for whom preoperative imaging agreed with the localization of a single diseased parathyroid and those with only ultrasound or scintigraphy positive for parathyroid localization underwent Mini-Invasive Video-assisted parathyroidectomy. In all cases, ioPTH assay was performed. The conversion to bilateral neck exploration was decided based on the drop in ioPTH. CaPTHUS score and the Wisconsin index (Win) were applied to the series. CaPTHUS score ≥3 and Win index >1600, according to the original studies of the literature, were considered at high probability of monoglandular disease. Outcomes in these two groups were examined.

**Results:**

236 patients were eligible for the study. The pathology resulted in multiglandular disease in 24 patients (10.2%). Among these, 18 (75.0%) obtained a CaPTHUS score ≥3, and 20 (83.3%) had a Win index>1600. Intraoperative PTH allowed to identify multiglandular disease in 16 of 18 cases with CaPTHUS ≥3 and in 18 of 20 cases with win >1600, who could have been lost, based only on the results of these 2 tests.

**Conclusion:**

Based on our experience, CaPTHUS test and Wisconsin index were not so useful in predicting multiglandular disease as ioPTH.

## 1. Introduction

Primary hyperparathyroidism (PHPT) is a common endocrine pathology with a prevalence of 1 per 1,000 in men and 2–3 per 1,000 in women [1]. PHPT is caused by a single-gland adenoma in 80–85% of the cases. In 15–20%, it is due to the involvement of more parathyroid glands, either in the form of hyperplasia of all glands or in the form of multiple adenomas [2]. Parathyroid carcinoma is rare and accounts for less than 1% of the cases [2]. The only definitive treatment for PHPT is surgical therapy [3]. The ability to correctly identify if the source of the gland's hyperfunction is a single adenoma or a multiglandular disease is essential for the success of the surgical treatment. The traditional surgical approach involves the identification of all parathyroid glands through bilateral neck exploration (BNE), and the removal of the macroscopically pathological ones [4]. Due to the improvement of localization exams and the addition of intraoperative tests, such as intraoperative parathormone assay (ioPTH) [5], currently focused parathyroidectomy (FP) is the most used approach for the surgical treatment of PHPT. Indications for FP are the accurate preoperative localization of a single pathological gland by imaging investigations, no previous neck interventions, and the absence of associated thyroid pathologies. When performed, success rates comparable to those of the traditional approaches are achieved, while the risk of complications, postoperative pain, operative time, length of hospital stays, and hospital costs are all reduced [6–10]. To perform FP, it is important to verify whether all pathological tissues were removed or not. Intraoperative PTH has proven to be a reliable method in predicting the eradication of the disease [11]. However, ioPTH is not available in all centers, and it increases the cost of treatment and operating time [12, 13]. For these reasons, numerous studies have been conducted to identify a preoperative score that would allow surgeons to predict the presence of multiglandular disease, to establish the most suitable surgical approach. The most frequently examined were CaPTHUS score, described by Kebebew et al. in 2006 [14], and Wisconsin index (Win score), described by Mazeh et al. in 2013 [15]. The aim of our study is to verify the utility of these two preoperative scores in predicting single-gland disease, through the analysis of our center experience.

## 2. Materials and Methods

This is a retrospective cohort study on 462 consecutive adult patients referred for parathyroidectomy to the Thyroid and Parathyroid Unit at Santi Paolo *e* Carlo Hospital, Milan, Italy, from 2011 to 2021. All cases were retrieved from a Microsoft Access Database (Version 2001, Microsoft Corp, Redmond, WA, US), where patients are registered after discharge. Only patients with benign PHPT were included in the study. Exclusion criteria were as follows: persistent or relapsed primary hyperparathyroidism and follow-up of less than 6 months. The diagnosis of PHPT was made based on calcium, phosphorus, PTH and vitamin D 25-OH blood levels, and 24-hour urine calcium. All patients, after diagnosis, underwent neck ultrasound and ^99^Tc-labeled sestamibi scintigraphy (MIBI). To eliminate a possible bias, since the accuracy of the ultrasound scans in locating a pathological parathyroid is linked to the expertise of the operator, in this study, we included only patients who underwent ultrasound by an experienced radiologist from our hospital. Both patients for whom preoperative imaging agreed on the localization of a single diseased parathyroid and those with only ultrasound or scintigraphy positive for parathyroid localization underwent parathyroidectomy with the Mini-Invasive Video-assisted Parathyroidectomy (MIVAP) technique; when ultrasound and scintigraphy were discordant, we based upon ultrasounds of the expert radiologist. In all cases, an intraoperative PTH assay was performed. The conversion of the procedure to the traditional bilateral cervical exploration was decided based on the drop in the ioPTH. In all patients, ioPTH was evaluated 10 minutes after parathyroidectomy, and if there was a drop in ioPTH values greater than 50% compared to the baseline (Vienna criteria), the surgery was considered concluded. When the drop in PTH 10 minutes after parathyroidectomy was less than 50%, a second sampling was performed to evaluate the possibility of late drop of the hormone. If the failure to the drop of PTH was confirmed, a traditional bilateral cervical exploration was carried out.

The following data were considered in the evaluation of patients: demographic data (age at surgery and sex), preoperative biochemical data (PTH, calcium, vitamin D, and urinary calcium), preoperative imaging (ultrasound and scintigraphy), ioPTH, histological outcomes, glands weight, and outcome of surgery (follow-up of at least 6 months). PTH values are expressed in pg/mL (normal range 8.7–79.6 pg/mL) and serum calcium values in mg/dL (normal range 8.4–10.2 mg/dL). Persistent hyperparathyroidism was defined as hypercalcemia that develops immediately after surgery or within 6 months of it. Recurrent hyperparathyroidism was defined as the development of hypercalcemia in a patient who was hypocalcemic or normocalcemic for at least 6 months after parathyroidectomy. Patients were followed up by reevaluating calcemia, PTH, and vitamin D-25-OH at 3 months, 6 months, and 1 year after surgery.

The CaPTHUS test and the Wisconsin index (Win) were applied to the series.

The CaPTHUS test is characterized by 5 conditions, each of which is assigned a score of 0 or of 1. The score is 1 if the condition is satisfied; otherwise, it is 0. The sum of each of these scores results in the value of the CaPTHUS score, which can range from a minimum of 0 to a maximum of 5. The conditions considered are described and shown in Table 1.

With a score of 5, the patient possesses all characteristics taken into consideration. With a minimum value of 0, the patient does not have any of the listed values. A CaPTHUS value ≥ 3 should be indicative of single-gland disease.

The Wisconsin index (Win score) takes into consideration the preoperative calcium and parathyroid hormone values, which must be multiplied to obtain the score. Patients are then divided into three classes: Win scores equal or greater than 1600, between 1600 and 800, and equal or less than 800. It is hypothesized that patients with multiglandular disease have significantly low win values. The Win score classes are shown in Table 2.

Sensitivity (Sn), specificity (Sp), positive predictive value (PPV), negative predictive value (VPN), and, finally, accuracy were evaluated for the proposed tests. In addition, confidence intervals (CI) were calculated. Specificity, sensitivity, positive and negative predictive values, and prevalence are expressed in percentages. Confidence intervals for specificity, sensitivity, and accuracy were calculated according to the Clopper–Pearson method.

## 3. Results

Among the 462 patients, 236 were eligible for analysis. Among these, 184 were female (78.0%) and 52 men (22.0%), and the median age was 64 years (with a range 22–85, average of 61.4, and SD ± 12.3). Of these, 212 patients (89.8%) underwent MIVAP, and the remaining 24 patients (10.2%) underwent bilateral neck exploration. 13 patients also underwent a thyroidectomy during the same operating session.

For each patient, two localization imaging techniques were considered: ultrasound and ^99^Tc-labeled sestamibi scintigraphy (MIBI).

All 236 patients underwent neck ultrasounds. It revealed only one enlarged gland in 205 cases (86.9%); in the remaining 31 (13.1%), it was not possible to identify any enlarged parathyroid.

All 236 patients underwent scintigraphy; 200 (84.7%) were found positive in locating a single hyperfunctioning gland, and the remaining 36 (15.3%) were negative.

Overall, 200 patients (84.7%) had concordant imaging, while, in 36 cases, ultrasound and scintigraphy did not indicate the same side (15.3%).

Clinical features of the patients are shown in Table 3.

The histological findings were divided as follows: single-gland disease in 212 patients (89.8%) and multiglandular in 24 (10.2%). In particular, the definitive diagnosis was as follows: single adenoma for 212 patients (89.8%), hyperplasia for 21 (8.9%), and double adenoma for 3 (1.3%). MEN1 was diagnosed in 3 patients with hyperplasia.

Patients treated for PHPT caused by parathyroid carcinoma (PC) were excluded, because they showed average calcium and PTH preoperative values higher than patients treated for benign PHPT. Moreover, they always had a preoperative concordant localization, since PC is generally a monoglandular disease, with a larger diameter than adenoma. All these aspects could have been a bias for the study, considering that all above-mentioned parameters are considered in calculating the CaPTHUS score and the Wisconsin index.

Histological features of all patients are shown in Table 4.

Overall, considering the 236 patients enrolled in the study, 6 had a relapse or persistence of disease (2.5%), with an intervention success rate of 97.5%, considering these selected series, which is comparable to 97.8% if we refer to all patients treated at our center for Primary Hyperparathyroidism, in the same period. Of these 6 patients, only the data relating to the first intervention were included for the study, and details about them are reported at the end of the results section.

All patients were followed up after surgery by reevaluating calcium, parathyroid hormone, and vitamin D-25-OH 3 months, 6 months, and 1 year after the intervention.

The patients were then divided into two groups: single-gland disease (adenoma) and multiglandular disease (hyperplasia and double adenoma).

The group of patients with single-gland disease consisted of 212 patients (89.8%); 166 (78.3%) were women, and 46 (21.7%) were men; the average age was 61.6 years.

The group of patients classified as multiglandular disease was made up of 24 subjects (10.2%), of which 18 are females (75.0%) and 6 are males (25.0%); in this group, the average age was 59.3 years. The values of calcemia and parathyroid hormone and, furthermore, the results of the diagnostic imaging are summarized in Table 5 (single-gland) and Table 6 (multiglandular).

Intraoperative PTH assay was performed for all patients. The sampling of peripheral blood was performed at the induction of anaesthesia and 10 minutes after the removal of the pathological parathyroid. Once the gland was removed, any manoeuvres were stopped for 10 minutes before sampling, to avoid artefacts due to local manipulation. The basal sample and the sample 10 minutes after the excision of the gland were sent to the central laboratory for the analysis of the parathyroid hormone. A reduction of more than 50% in parathyroid hormone values was considered as an indication of the success of the intervention. When the parathyroid hormone descent was not significant, a third sampling was performed. If the parathyroid hormone in the third sampling was nondescent, then it was considered indicative of multiglandular disease, and the intervention was converted into a bilateral exploration.

The average of the baseline PTH values was 289.04 ± 304.69; the median of the values was 205.85. For PTH values at 10 minutes, the mean was 55.34 ± 73.05 with a median of 35.55.

The CaPTHUS test was applied to the 236 patients, who entered the study and were then divided into 6 classes, shown in Table 7.

According to the original study of Kebebew, we divided the patients in two groups: patients with a CaPTHUS score ≥3 (highest probability of monoglandular disease) and patients with CaPTHUS score <3 (highest probability of multiglandular disease). For 201 (85.2%) patients, the CaPTHUS score ≥3. Among these, 18 patients had the definitive diagnosis of multiglandular disease. We identified 16/18 of these cases through ioPTH assay and missed the other 2. For 35 (14.8%) patients, the CaPTHUS score <3, with 35 cases of adenoma and 6 of multiglandular disease. Among these 6 cases, 4 were identified by ioPTH, while the other two were missed (Table 8).

CaPTHUS score had a sensitivity of 13.68%, a specificity of 75.00%, a PPV of 82.86%, a NPV of 8.96%, and an accuracy of 19.92% (Table 9).

The Win test was applied to the 236 patients recruited, who were thus distributed in 3 classes: 179 (75.8%) patients in the class with a score >1600, 56 (23.8%) in the class with the score 800–1600, and 1 (0.4%) score <800 (Table 10).

Among 179 patients with Win index >1600, considered with high probability of monoglandular disease, according to the original study of Mazeh et al., 20 were multiglandular. Among these, 18/20 (90%) were diagnosed, thanks to ioPTH assay, while 2 were missed (Table 11).

The Win score had a sensitivity of 75.0%, a specificity of 16.7%, a PPV of 88.8%, a NPV of 7.1%, and an accuracy of 69.1% (Table 12).

## 4. Persistence/Relapse

Among 6 patients with persistence/recurrence of PHPT, 5 showed persistence at blood tests 3 three months after the intervention, and 1 patient developed relapse 8 months after the intervention. This patient had a relapse after inferior left MIVAP, with significative ioPTH drop. This patient was a 62-year-old male, with CaPTHUS >3 and Win score 3. The histological diagnosis was adenoma. After the relapse, neck ultrasound and scintigraphy showed an enlarged inferior right gland. He underwent inferior right parathyroidectomy 12 months after the first intervention, with significative i.o. PTH drop. Histological diagnosis was of adenoma. He has normal serum calcium and PTH 60 months after the second intervention.

2 of the 5 patients with persistence underwent reintervention. 1 of these was a 60-year-old female with severe osteoporosis, CaPTHUS 1 and Win score >3. She had persistence three months after the removal of superior right, inferior right and superior left parathyroid, and thyroidectomy for goiter. Both calcium and PTH levels were in the range at when she was discharged from hospital. Histological diagnosis was hyperplasia of both parathyroid and thyroid. After the diagnosis of persistence neck ultrasounds was negative, scintigraphy was positive, with uptake in the left side of the neck. Because of severe osteoporosis, indication for reoperation was given. She underwent inferior left radio-guided parathyroidectomy 9 months after the first intervention, with histological confirmation of hyperplasia. In the immediately postoperative period, she had normal values of PTH and calcium, despite the removal of 3 glands in the first intervention and 1 in the second. Then, a scintigraphy was performed without the evidence of further hyperfunctioning parathyroid tissue. A CT scan of neck and chest was negative for ectopic glands. The patient is in follow-up and 23 months after the reintervention has normal serum calcium and PTH at the upper level.

The other one who underwent reintervention, 6 months after the first operation, was a 32-year-old female with preoperative neck ultrasound and scintigraphy concordant in locating an enlarged inferior left parathyroid. She had CaPTHUS >3 and Win 3. At the first intervention, there was the confirmation of an enlarged inferior left gland. It was removed, without a significative ioPTH drop, with histological intraoperative diagnosis of “hypercellular parathyroid.” At further exploration, the superior left gland was enlarged. It was removed with a significative ioPTH drop. For this reason, the right side was not explored. Histological definitive diagnosis was double adenoma (inferior and superior left). Calcium and PTH were normal for 3 months after the intervention, and then the patient showed a persistence. Imaging (ultrasounds, scintigraphy, and TC scan of neck and chest) was negative. Because of her age and symptoms, she underwent exploration of the right side of the neck, 6 months after the first intervention. The inferior right parathyroid was enlarged and behind the jugulum, while the superior is in the range of normality. Inferior right parathyroid was removed with significative ioPTH drop. The specimens of the two glands removed at first intervention and the inferior right gland were reviewed together by the same expert pathologist, with the diagnosis of hyperplasia on all the three removed glands. MEN1 was subsequently diagnosed. She has normal calcium and PTH values after 96 months.

Among the 3 patients who did not undergo reintervention, 1 is a 56-year-old woman with osteoporosis, CaPTHUS >3, and Win 3, who underwent inferior left MIVAP with significative ioPTH drop and histological diagnosis of inferior left adenoma. She showed persistence at blood tests 3 months after the intervention. The scintigraphy showed hyperfunctioning tissue in the mediastinum. A CT scan confirmed a lesion in the upper posterior mediastinum, compatible with an ectopic parathyroid gland. The patient refused reintervention.

For the other 2 patients, a 58-year-old male affected with AIDS and a 72 old-year-old female, it was decided to continue with the follow-up, in consideration of comorbidities for the male and the mild hyperparathyroidism for the female. Both showed the persistence 3 months after the intervention, had CaPTHUS >3 and a WIN score 3, and underwent bilateral neck exploration at first intervention, due to the failure of the ioPTH descent, with the removal of three glands and histological diagnosis of hyperplasia.

## 5. Discussion

Surgery still represents the best treatment for PHPT, compared to observation or medical therapy. It allows to cure the disease definitively, avoiding the possible side effects of drugs and the additional costs that would be necessary, due to the need of clinical follow-up and laboratory tests [16].

The traditional surgical approach involves the median cervicotomy with bilateral cervical exploration, visualization of all four parathyroid glands, removal of the macroscopically pathological one, and the biopsy of the smaller ones, subjected to extemporaneous histological examination [17].

Focused unilateral access involves a mini-cervicotomy with direct access to the pathological gland, identified by imaging and intraoperative dosing of PTH. This approach limits the surgical exploration to only one side in the presence of a single-gland disease, avoiding the devascularization of the nonpathological glands and reducing the risk of hypoparathyroidism. It also limits the exposure of the inferior laryngeal nerve to only one side, reduces the operating times, and allows a better aesthetic result, with less postoperative pain [18].

About the treatment, both the traditional and the focused technique had the same results, with a success rate of 98% of cases in highly specialized centers [6, 10], comparable to the 97.5% obtained at our institution. In our experience, the failure of surgery was due in 3 cases to a false drop of IOPTH. In one of these, the patient had the diagnosis of double adenoma, after the second intervention. This is a well-known cause of failure of ioPTH, which may be explained because one of the two adenomas would be more functioning than the other at the time of the first diagnosis. The second adenoma would start functioning within a variable period, only after the removal of the first [19]. A similar mechanism may explain the case of persistence for the patient who refused the reintervention, for whom an adenoma was removed in the neck and after three months showed a persistence. This was due to an ectopic gland, which emerged only after the removal of the gland in the neck. About the other cases of persistence, 1 was due to supernumerary glands, and 2 to an insufficient resection at first intervention. In both cases, the multiglandular disease was correctly diagnosed, and three glands were removed.

The introduction of minimally invasive methods has led to an increasing demand for focused parathyroidectomy in the surgical treatment of PHPT. This approach requires verifying the adequacy of the intervention, which is why the use of intraoperative dosage of PTH has begun since the end of the 1990s [19]. Since this examination is not available in all centers, especially because of the additional costs, some authors have sought models that would allow the prediction of single-gland pathology, to use minimally invasive surgical procedures, even without having ioPTH available. In 2006, Kebebew et al. proposed the CaPTHUS test [14], which allows to combine biochemical and diagnostic imaging data, to select patients with a high probability of single-gland disease. The study proposed as cut-off score 3: according to this, only in case of a CaPTHUS score test <3, it was indicated to perform ioPTH. In these cases, the test provided a good estimate of the probability of multiglandular disease, with a precision of almost 100%. The original work [14], which proposes the CaPTHUS test, demonstrated the correlation between biochemical and diagnostic imaging tests and single-gland disease but presented some limitations. It was conducted by excluding all subjects who had not performed both imaging methods and considering as curing all patients who had normal calcium levels one week after surgery. The failure to evaluate patients after adequate follow-up did not allow to establish the actual healing of the subjects, classifying even those who have relapsed over time as single-gland diseases.

In 2015, Elfenben et al. [20] and, the following year, Mogollon-Gonzalez et al. [21] conducted two studies with the aim of validating the CaPTHUS score. The first [20] included, in the evaluation of CaPTHUS score, also patients who had undergone only scintigraphy or only neck ultrasound. In this way, with a score of ≥3, the PPV decreased to 91% in predicting single-gland disease. Moreover, without measuring ioPTH for patients with high CaPTHUS scores, the cure rate was 89% at 6 months, compared with 98% when the assay was performed. In the second study, Mogollon-Gonzalez et al. [21] chose a follow-up of at least six months but decided to include only patients who had performed both tests. In this case as well, the CaPTHUS test was found to be a model that even though it accurately predicts multiglandular disease, it does not allow to correctly classify 100% of patients, therefore allowing to reduce the use of ioPTH.

In our study, the pathology showed multiglandular disease in 24 patients (10.2%). Considering the CaPTHUS test, among these 24 patients, 13 (54.2%) had a CaPTHUS >3. If ioPTH had not been performed in patients with CaPTHUS >3, as suggested by Kebebew [14], 54.2% (13/24) of all patients affected with multiglandular disease, instead of 8.3% (2/24) obtained by performing ioPTH assay in this group, would not have been cured. We would have obtained an even worse result if we had used the Win Index: among 24 patients affected with multiglandular disease, 20 (83.3%) achieved a score ≥1600. If ioPTH had not been performed in these patients, 83.3% of all patients affected with multiglandular disease, instead of 8.3% by performing ioPTH, would have not been cured.

The Win score was proposed by Mazeh et al. in 2013 [15], aiming to predict multiglandular disease. It takes into consideration only the biochemical parameters, in particular calcium and parathyroid hormone, to classify patients: subjects with elevated calcium and parathyroid hormone are more likely to present a single-gland disease. In our case series, however, there was no correlation between the levels of calcium and PTH and the underlying disease of PHPT. Similarly, in the literature [15], the win score allows to predict the risk of multiglandular disease only in relation to the weight of the gland removed, although laboratory data alone is not a sufficient indication. In our series, which has the limit of being a retrospective study, the unsatisfactory results in predicting single-gland disease by two scores can be attributed to the fact that both the CaPTHUS test and the win score use the same biochemical data. Only 41.5% of patients had levels of Ca and PTH high enough to satisfy both parameters of the CaPTHUS test. This may be due also to the fact that, in the last years, we observed a change in the presentation of PHPT, from a clinical and biochemical point of view. Normocalcemic hyperparathyroidism with surgical indication is increasingly common [2, 22, 23]. In our series, by comparing the preoperative scores with ioPTH, it can be noticed that the models studied are not as effective in predicting the presence of multiglandular disease as intraoperative monitoring of PTH. Even when selecting subjects with a high score at CaPTHUS test (4 and 5 or only 5) and at Wisconsin index (>1600), the drop in intraoperative PTH has been more reliable, allowing to avoid a greater number of nondefinitive treatments.

## 6. Conclusion

Based on our experience, the CaPTHUS score and the Wisconsin Index have not been proved preoperatively so useful in distinguishing between mono- and multi-glandular disease, as ioPTH assay. Intraoperative tests confirmed their accuracy in predicting the adequacy of the intervention, and the patient has been cured. For this reason, in cases of a focused approach to parathyroid diseases, it is advisable to perform ioPTH dosage, regardless of the patients' preoperative characteristics.

## Figures and Tables

**Table 1:** CaPTHUS score.

Total serum calcium	≥12 mg/dL

PTH	≥2 times then the upper limit
Ultrasound	Positive for one enlarged gland
Scintigraphy	Positive for one enlarged gland
Imaging (ultrasound + scintigraphy)	Concordance

**Table 2:** Win score (Ca x PTH).

≥1600	High
1600–800	Medium
≤800	Low

**Table 3:** Clinical features of patients with PHPT (*n* *=* 286).

Clinical features	Value
*Sex n (%)*	
Female (F)	184 (78.0%)
Male (M)	52 (22.0%)
*Age at surgery*	
Mean ± SD	61.4 ± 12.3
Median (range)	64 (22–85)
*Preoperative serum calcium (mg/dL)*	
Mean ± SD	12.08 ± 1.31
Median (range)	11.9 (9.34–19.24)
*Preoperative PTH*	
Mean ± SD	311.4 ± 301.93
Median (range)	206.7 (71.4–1910.0)
*Ultrasound*	
Positive	205 (86.86%)
Negative	31 (13.14%)
*Scintigraphy*	
Positive	200 (84.7%)
Negative	36 (15.3%)
*Concordant imaging*	
Yes	200 (84.7%)
No	36 (15.3%)

**Table 4:** Definitive diagnosis and success of surgery.

Clinical feature	Value
*Histological finding n (%)*	
Single adenoma	212 (89.8%)
Double adenoma	3 (1.3%)
Hyperplasia	21 (8.9)
*Parathyroid weight (mg)*	
Mean ± SD	(i) 2011.5 ± 4463
Median (range)	(ii) 1000 (30–56000)
Multiglandular disease *n* (%)	24 (10.2%)
Single-gland disease *n* (%)	212 (89.8)
Persistence or relapse *n* (%)	6 (2.5%)

**Table 5:** Clinical features of patients with single-gland disease (*n* *=* 212).

Clinical features	Value
*Sex n* (%)	
Female (F)	166 (78.3%)
Male (M)	46 (21.7)
*Age at surgery*	
Mean ± SD	61.65 ± 12.40
Median (range)	64 (22–85)
*Preoperative serum calcium (mg/dL)*	
Mean ± SD	12,10 ± 1.25
Median (range)	11.97 (9.34–19.24)
*Preoperative PTH*	
Mean ± SD	304.80 ± 289.12
Median (range)	198.6 (71.4–1878)
*Imaging N*	
Positive ultrasound	195
Positive scintigraphy	180
Concordant imaging	189
*Parathyroid weight (mg)*	
Mean ± SD	1578.31 ± 2024.28
Median (range)	1000 (70–20000)

**Table 6:** Clinical features of patients with multiglandular disease (*n* *=* 24).

Clinical features	Value
*Sex n (%)*	
Female (F)	18 (75.0%)
Male (M)	6 (25.0%)
*Age at surgery*	
Mean ± SD	59.28 ± 11.37
Median (range)	660 (32–76)
*Preoperative serum calcium (mg/dL)*	
Mean ± SD	12.07 ± 1.82
Median (range)	11.55 (10.4–18.0)
*Preoperative PTH*	
Mean ± SD	369,77 ± 400.82
Median (range)	236 (99–1910)
*Imaging N*	
Positive ultrasound	10
Positive scintigraphy	20
Concordant imaging	11
*Parathyroid weight (mg)*	
Mean ± SD	5838.04 ± 12205.80
Median (range)	1500 (30–56000)

**Table 7:** CaPTHUS score.

CaPTHUS		Absolute frequency
CaPTHUS 0	Total	7 (2.96%)
	Multiglandular	2
	Single-gland	5

CaPTHUS 1	Total	13 (5.51%)
	Multiglandular	1
	Single-gland	12

CaPTHUS 2	Total	15 (6.35%)
	Multiglandular	3
	Single-gland	12

CaPTHUS 3	Total	58 (24.57)
	Multiglandular	5
	Single-gland	53

CaPTHUS 4	Total	82 (34.75)
	Multiglandular	9
	Single-gland	73

CaPTHUS 5	Total	61 (25.85%)
	Multiglandular	4
	Single-gland	57

**Table 8:** CaPTHUS score and ioPTH descent after excision of a single gland.

CaPTUS	Disease		ioPTH descent	
			>50%	<50%
0–2	Multiglandular	6	0	6
(*n* *=* 35)	Single-gland	29	27	2

3–5	Multiglandular	18	2	16
(*n* *=* 201)	Single-gland	183	180	3

**Table 9:** CaPTHUS.

CaPTHUS	Single-gland		Multiglandular		Total
CaPTHUS 0–2	29		6		35
CaPTHUS 3–5	183		18		201
	212		24		236

	Sensitivity	Specificity	PPV	NPV	Accuracy
	13.68%	75.00%	82.86%	8.96%	19.92%
95% CI	9.36% to 19.05%	53.29% to 90.23%	69.09% to 91.27%	7.20% to 11.09%	15.02% to 25.59%

**Table 10:** Win index (Ca*∗*PTH).

>1600	Total	179 (75.8%)
	Multiglandular	20
	Single-gland	159

1600–800	Total	56 (23.8%)
	Multiglandular	4
	Single-gland	52

<800	Total	1 (0.4%)
	Multiglandular	0
	Single-gland	1

**Table 11:** Win score and ioPTH after excision of a single gland.

WIN	Disease		ioPTH descent	
			>50%	<50%
1	Multiglandular	0	0	0
(*n* *=* 1)	Single-gland	1	1	0
2	Multiglandular	4	0	4
(*n* *=* 56)	Single-gland	52	50	2
3	Multiglandular	20	2	18
(179)	Single-gland	159	157	2

**Table 12:** Win score.

Win	Single-gland		Multiglandular		Total
≥1600	159		20		179
<1600	53		4		57
Tot	212		24		236

	Sensitivity	Specificity	VPP	VPN	Accuracy
	75.00%	16.67%	88.83	7.02%	69.07%
95% CI	68.61% to 80.68%	4.74% to 37.38%	86.74% to 90.62%	2.91% to 15.98%	62.75% to 74.90%

## Data Availability

All data were retrieved from a Microsoft Access Database (version 2001, Microsoft Corp, Redmond, WA, US), where patients are registered after discharge, and are available from the corresponding author upon request.
